# Re: Gaseous bladder tamponade secondary to emphysematous cystitis

**DOI:** 10.1590/S1677-5538.IBJU.2017.0701

**Published:** 2018

**Authors:** Yu-Chen Chen, Hao-Wei Chen, Yung-Shun Juan, Wen-Jeng Wu, Chia-Chun Tsai

**Affiliations:** 1 Department of Urology, Kaohsiung Medical University Hospital, Kaohsiung Medical University, Kaohsiung, Taiwan; 2 Graduate Institute of Clinical Medicine, College of Medicine, Kaohsiung Medical University, Taiwan; 3 Department of Urology, Kaohsiung Municipal Ta-Tung Hospital, Kaohsiung, Taiwan

*To the editor,*

Recently, Yang et al. (1) published the abdominal computed tomography (CT) images showing diffuse gas within the bladder wall and a prominent air-fluid level as the typical manifestation of emphysematous cystitis. However, it's not only involved in the bladder wall but also in the bladder lumen (2, 3). We hereby present a case of gaseous bladder temponade causing obstructive uropathy – a rarely severe complication of emphysematous cystitis.

Our patient is a 81-year-old man who presented to the emergency department with 2 days of history of fever, progressed low abdominal distention and decreased urine output. He had type 2 diabetes mellitus and flaccid neurogenic bladder with long-term indwelling Foley catheter for the preceding two years. Laboratory studies revealed bacteriuria, leukocytosis, an elevated C-reactive protein level, high fasting blood glucose (302mg/dL), and an elevated creatinine level (2.05mg/dL). Radiography of the kidneys, ureters, and bladder revealed a low density over the whole bladder area and linear collection of gas along bladder wall (Figure-1). Abdominal CT was subsequently arranged, which showed gas tamponade in the bladder with bilateral obstructive hydronephrosis (Figure-2). A new Foley catheter was changed, and bladder irrigation was performed to extract pus and gas. He was treated with broad-spectrum antibiotics and strict blood sugar control. Both blood and urine cultures grew Klebsiella pneumonia. He responded with defervescence and recovery of renal function (creatinine=1.39mg/dL) after a full course of antibiotics treatment.

**Figure 1:**
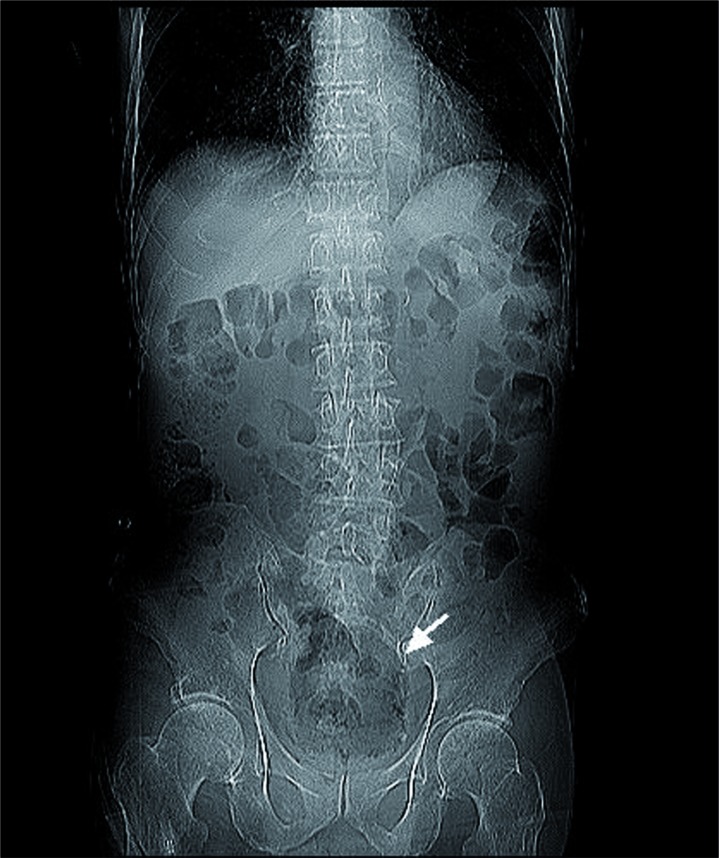
Radiography of the kidneys, ureters, and bladder revealed a low density over the whole bladder area and linear collection of gas along bladder wall (arrow).

**Figure 2:**
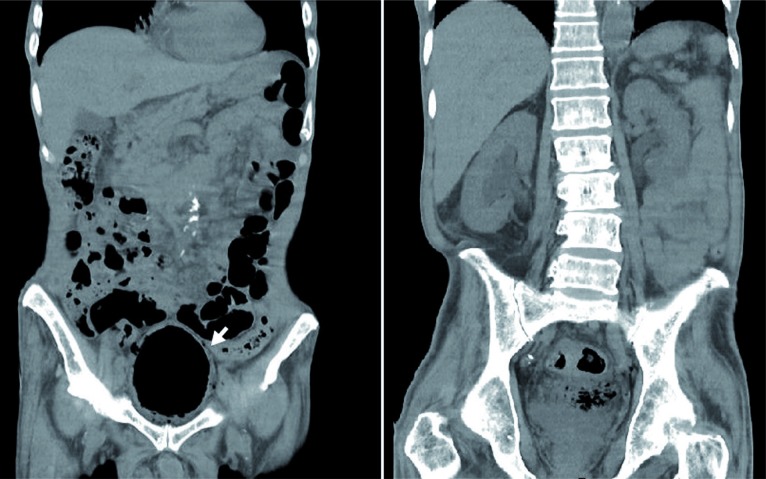
Abdominal computed tomography revealed bladder tamponade with massive air accumulation in bladder lumen and bladder wall (arrow), which leaded to bilateral hydronephrosis.

Emphysematous cystitis is an uncommon disease characterized by the presence of air within the bladder wall and lumen, and primarily observed in diabetic patients. In this case, gaseous bladder tamponade with bilateral obstructive hydronephrosis is a rarely emergency complication of emphysematous cystitis. Prompt diagnosis by radiography, broad-spectrum antibiotics, immediate drainage and intensively underlying diseases control are critical. Importantly, we suggest that all patients with long-term indwelling catheters and immunosuppression should note this rare sequela.

## References

[1] Yang Z, Sheng C (2017). Gas surrounding the urinary bladder in emphysematous cystitis. Int Braz J Urol..

[2] Quint HJ, Drach GW, Rappaport WD, Hoffmann CJ (1992). Emphysematous cystitis: a review of the spectrum of disease. J Urol..

[3] Grupper M, Kravtsov A, Potasman I (2007). Emphysematous cystitis: illustrative case report and review of the literature. Medicine (Baltimore)..

